# STAT1 deficiency supports PD-1/PD-L1 signaling resulting in dysfunctional TNFα mediated immune responses in a model of NSCLC

**DOI:** 10.18632/oncotarget.26441

**Published:** 2018-12-14

**Authors:** Juliane Friedrich, Lisanne Heim, Denis I. Trufa, Horia Sirbu, Ralf J. Rieker, Mircea T. Chiriac, Susetta Finotto

**Affiliations:** ^1^ Department of Molecular Pneumology, Friedrich Alexander University Erlangen-Nürnberg, Erlangen, Germany; ^2^ Department of Thoracic Surgery, Friedrich Alexander University Erlangen-Nürnberg, Erlangen, Germany; ^3^ Institute of Pathology, Friedrich Alexander University Erlangen-Nürnberg, Erlangen, Germany; ^4^ Department of Medicine 1-Gastroenterology, Pneumology and Endocrinology, Friedrich Alexander University Erlangen-Nürnberg, Erlangen, Germany

**Keywords:** NSCLC, STAT1, PDL1

## Abstract

In this study we described that Signal Transducer and Activator of Transcription 1 (STAT1) is a key point regulator of PD-1 in tumour infiltrating lymphocytes and PD-L1 in Tumour associated macrophages (TAM) in NSCLC. In our murine model of adenocarcinoma targeted deletion of Stat1 was found associated with enhanced tumour growth, impaired differentiation into M1-like macrophages from the bone marrow, the accumulation of tumor associated macrophages overexpressing PD-L1 and impaired T cell responses in the tumor microenvironment by affecting TNFα responses.

In our human NSCLC patient cohort we found that loss of isoforms STAT1 α and STAT1β mRNA in the tumoural region of the lung correlates with increased tumor size in NSCLC patients. Therefore, STAT1 isoform regulation could be considered for future therapeutical strategies associated to current immune-checkpoint blockade therapy in NSCLC.

## INTRODUCTION

The Programmed cell death 1 ligand 1, inhibitory ligand PD-L1, the ligand for the T cell inhibitory receptor PD-1, is a key mediator in regulating anti-tumor responses in human cancer patients as well as in different murine cancer disease model [1]. PD-L1 is expressed by various cancer cells in response to inflammatory cytokines [2]. Previous reports showed that PD-L1 is also expressed on tumor infiltrating macrophages (TAM) which is further on associated with a poor outcome of NSCLC patients [3]. Macrophages are important mediators in tumor defense by directly eliminating tumor cells [4] by secreting the reactive nitrogen species nitric oxide and TNFα. TNFα is a pleiotropic cytokine, which plays a dual role in the tumor microenvironment by controlling various functions such as apoptosis, invasion and proliferation [5]. In melanoma patients an increased TNF gene expression is seems to be linked to αPD-1 therapy [6]. Emerging immunotherapy strategies that block PD-1 or PD-L1 [7–9] have radically changed the fight against cancer. However, two third of the patients do not respond to αPD-1 therapy and in a significant proportion of patients treated with immune-checkpoint blockade (ICB) antibodies show relapse within two years after treatment induction [10–12]. Moreover, it has been reported that patients with tumors that express the PD-1 ligand prior to the treatment have a higher chance to respond to the treatment strategy [13] especially in the case of patients that suffered from NSCLC [14, 15]. Our group has previously defined STAT1 as an important key regulator in NSCLC, since we found a decreased activation of STAT1 in the tumoural region of patients that suffered from NSCLC compared to the tumor free control region [16]. STAT1 is described to play a key role in tumorigenesis by controlling a large variety of functions such as activating certain genes that block cell cycle progression or inhibiting angiogenesis [17]. Other groups have recently reported that JAK-STAT-mediated chronic inflammation impairs cytotoxic T lymphocyte activation to decrease anti-PD-1 immunotherapy efficacy in pancreatic cancer [18].

Here we directly evaluate the relative role of host STAT1 expression in immune cells and the PD-1/PD-L1 signaling axis in a murine model of lung adenocarcinoma. Loss of STAT1 in the host was found associated with increased tumour load, inhibition of the anti-tumour immune responses and by the upregulation of the immune suppressive markers PD-1 in TIL and PD-L1 in TAM as well as dysfunctional TNFα immune responses.

## RESULTS

### PD-1 blockade resulted in tendencial increased expression of *Stat1 mRNA*

Murine lung tumor development was induced by intravenously injection of LL/2-luc-M38 cells into the tail vein. At described days mice were treated intraperitoneally with 150 μg αPD-1 antibody or related isotype control (Supplementary Figure 1A). Similar to the previous described findings in humans [10–12] only half of the lung tumor bearing mice responded to the PD-1 blockade treatment [19]. Successful *in vivo* blockade of PD-1 was found associated with a tendencial upregulation of *Stat1* (Supplementary Figure 1B) and *Tbet* (Supplementary Figure 1C) mRNA expression measured in total lung cells derived from tumor bearing mice compared to tumor bearing mice treated with the related isotype control. Since no difference in the tumor development was detected, total lung cells from tumor bearing mice treated with blocking antibody or related isotype control were *in vitro* re-challenged for 24 h with 10 μg αPD-1 antibody or IgG2a, followed by qPCR analysis (Supplementary Figure 1D). *In vivo* blockade of PD-1 dominated the *in vitro* re-challenge. *Stat1, STAT2, STAT3* (Supplementary Figure 1E–1G) and *Tbet* mRNA expression (Supplementary Figure 1H) were tendencially upregulated in total lungs cells derived from αPD-1 antibody- treated tumor bearing mice independent from *in vitro* re-challenge.

### Loss of isoforms *STAT1α* and *STAT1β* mRNA in the tumoural region of the lung correlates with increased tumor size in NSCLC patients

**Figure 1:**
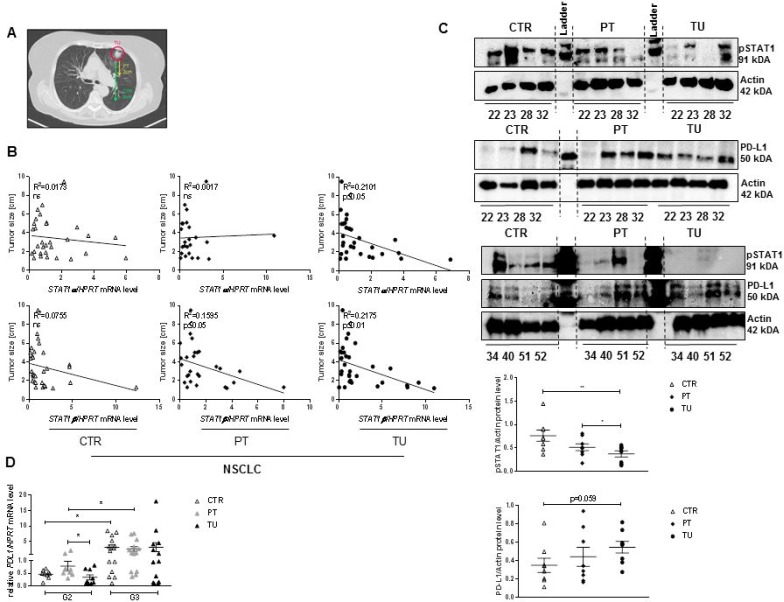
Dysfunction of STAT1 in patients that suffered from NSCLC and upregulation of the oncogene PD-L1 (**A**) Definition of analyzed tissue samples derived from patients that suffered from NSCLC. Tumoural region (TU), the solid tumor; peri-tumoural region (PT), tissue samples 2–3 cm away from the solid tumor; control region (CTR), tumor free region, at least 5 cm away from the solid tumor. (**B**) Decreased *STAT1*α mRNA, measured in the TU region of patients that suffered from NSCLC is associated with enlarged tumor size. No correlation between *STAT1*α mRNA level and tumor size were detected in the CTR and PT region. Impaired expression level of *STAT1β* mRNA is associated with enlarged tumor size in the CTR, PT and TU region of patients that suffered from NSCLC (n_CTR_ = 31, n_PT_ = 24, n_TU_ = 30). (**C**) In the tumoural microenvironment a significantly reduced activation of STAT1 (pSTAT1) was detectable on protein level. Impaired activation of pSTAT1 is associated with a slightly increase on PD-L1 on protein level in the tumoural region of patients that suffered from ADC (n_CTR_ = 8, n_PT_ = 8, n_TU_ = 8). (**D**) Upregulated *PDL1* mRNA in patients that suffered from NSCLC is associated with advanced tumor stage from G2 to G3 (n_CTR G2_ = 9; n_PT G2_ = 9; n_TU G2_ = 9; n_CTR G3_ = 14; n_PT G3_ = 13; n_TU G3_ = 13). Data are presented as mean values ± SEM; unpaired *t*-test ^*^p < 0.05, ^**^p < 0.01, ^***^p < 0.001.

We next wanted to determine if STAT1 is linked to tumor development in NSCLC patients. Our cohorts of patients recently described [16] include Adenocarcinoma (ADC) and Squamous cell carcinoma patients (SCC), collectively grouped as NSCLC patients (Table 1). For our studies we perform protein and mRNA analysis from tissue samples derived from the tumor itself (solid tumor, TU), the peritumoural area (PT, 2 cm away from the tumor) and a tumor free control region (CTR, at least 5 cm away from the tumor) (Figure 1A). STAT1 can be differentiated in STAT1α, the predominantly active pro-apoptotic form and STAT1β, which is able to modulate the effects of STAT1α [20, 21]. In our previous work we reported decreased phosphorylation, activation, of the STAT1α but not STAT1β isoform at protein level, in the tumoural region of the lung of our cohort of patients with ADC [21]. In the present study, we correlated both *STAT1* mRNA isoforms, expressed in the CTR, PT and TU region with the tumor size. Upregulated *STAT1α* mRNA was found associated with smaller tumor size in the TU region, whereas no correlation was found for *STAT1α* in the PT and CTR region from patients that suffered from NSCLC. Notably, upregulation of the *STAT1β* isoform correlated with reduced tumor size in all three regions of patients that suffered from NSCLC (Figure 1B). We next asked, whether reduced *STAT1* mRNA expression and big tumor size is linked to dysfunctional STAT1 activation. Therefore, the activated form of STAT1 (pSTAT1) was measured via Western Blot analysis. Indeed, we found reduced pSTAT1 in the TU compared to the CTR region in patients that suffered from NSCLC. In the same protein samples, PD-L1 protein expression was found upregulated in the TU region compared to the CTR region (Figure 1C). We next analysed *PDL1* mRNA expression in a bigger cohort of patients. Here we found that increased *PDL1* mRNA expression in the group of NSCLC patients with more severe tumor grade (Figure 1D).

**Table 1:** Clinical parameters of patients that suffered from lung adenocarcinoma

Sample ID	Histological Classification	Maximal tumor diameter [cm]	Grading	T	N	M	TNM Stadium	Gender	Age	Average Smoking (P/Y)
1-MP	SCC	1,3	G3	1b	0	0	IA	Male	80	40
2-MP	SCC	5,1	G3	1a	0	0	IA	Male	57	40
3-MP	ADC	5	G3	2a	0	0	IB	Male	79	60
4-MP	SCC	2	G3	1b	1	0	IIA	Female	53	25
5-MP	SCC	10,5	G2	3	0	0	IIB	Female	67	50
8-MP	SCC	5,5	G3	3	0	0	IIB	Male	66	30
9-MP	ADC	2,7	G2	1b	2	0	IIIA	Female	84	0
13-MP	SCC	3	G3	1b	0	0	IA	Male	69	50
14-MP	SCC	1,9	G2	1a	0	0	IA	Female	58	30
15-MP	ADC	2,5	G3	1b	0	0	IA	Male	63	100
16-MP	ADC	4,6	G3	3	0	0	IIB	Female	70	15
17-MP	ADC	2,6	G2	2	0	0	IB	Male	74	70
19-MP	ADC	6,5	#	2b	0	0	IIA	Female	55	30
21-MP	SCC	2,5	G1	1b	0	0	IA	Male	41	10
22-MP	ADC	7	G3	2b	1	0	IIB	Male	68	82
23-MP	ADC	4,5	G2	2a	0	0	IB	Male	73	75
26-MP	ADC	1,3	G3	1a	0	1	IV	Female	52	50
27-MP	ADC	1,4	G3	1a	0	0	IA	Female	70	50
28-MP	ADC	1,2	G3	1a	0	0	IA	Male	76	60
29-MP	SCC	3,7	G3	1b	0	0	IIB	Male	74	100
30-MP	SCC	1,8	G3	1a	0	0	IA	Female	70	30
32-MP	ADC	4,4	G3	2a	2	0	IIIA	Female	60	30
34-MP	ADC	1,8	G3	1	0	0	IA	Female	51	45
35-MP	ADC	3	G3	1b	0	0	IA	Female	72	0
36-MP	SCC	3,5	G3	2a	1	0	IB	Male	74	40
37-MP	SCC	3,3	G2	2a	1	0	IIA	Male	60	45
41-MP	SCC	1,3	G3	3	1	0	IIIA	Male	70	42
42-MP	SCC	1,5	G3	2b	1	0	IIB	Male	74	40
40-MP	ADC	1,8	G3	1a	1	0	IIA	Male	82	100
43-MP	ADC	4	G3	2a	2	0	IIIA	Female	72	0
46-MP	SCC	9,5	G3	3	1	0	IIIA	Male	60	30
47-MP	SCC	6	G3	2b	0	0	IIA	Male	64	80
48-MP	SCC	5	G2	2b	0	0	IIA	Male	55	20
50-MP	SCC	2,5	G3	3	1	0	IIIA	Male	70	0
51-MP	ADC	2,4	G3	1b	2	1	IV	Male	62	90
53-MP	ADC	2,25	G2	1a	0	0	IA	Male	62	10
54-MP	SCC	4,1	G2	3	0	0	IIB	Male	72	0
55-MP	ADC	1,8	G3	1a	2	0	IIIA	Female	64	40
56-MP	ADC	4	G2	2a	0	0	IB	Female	67	0
57-MP	ADC	3,8	G2	2a	0	0	IB	Female	35	10
58-MP	ADC	6,5	G3	3	0	0	IIB	Female	69	0
59-MP	ADC	0,9	G2	4	0	0	IIIA	Male	70	#
60-MP	SCC	2,5	G2	1b	1	0	IIA	Male	71	#
61-MP	SCC	1,1	G2	1a	0	0	IA	Male	75	#
62-MP	ADC	3,5	G2	1b	0	0	IA	Female	80	#
62-MP	ADC	3,5	G2	1b	0	0	IA	Female	80	#
63-MP	SCC	9	G3	3	1	0	IIIA	Male	69	#
64-MP	ADC	3,5	G2	1b	0	0	IA	Male	55	35
65-MP	SCC	2,8	G3	1b	0	0	IA	Female	76	#

Abbreviations: ADC=Adenocarcinoma, SCC=Squamous Cell Carcinoma, G2=moderately differentiated; G3=poorly differentiated, T-primary tumor: 0=no evidence of primary tumor; 1a=tumor 2 cm or less in greatest dimension; 1b=tumor more than 2 cm but not more than 3 cm in greatest dimension; 2a=tumor more than 3 cm but not more than 5 cm in greatest dimension; 2b=tumor more than 5 cm but not more than 7 cm in greatest dimension; 3=tumor more than 7 cm. N-regional lymph nodes: 0=no regional lymph node metastasis; 1=metastasis in ipsilateral peribronchial and/or ipsilateral hilar lymph nodes and intrapulmonary nodes, including involvement by direct extension; 2=Metastasis in ipsilateral mediastinal and/or subcarinal lymph node(s). M-distant metastasis: 0=no distant metastasis; 1=distant metastasis, # means not specified.

### *In vivo* blockade of PD-L1 is associated with upregulation of PD-1 in tumor infiltrating lymphocytes (TIL) in a murine model of lung adenocarcinoma

**Figure 2:**
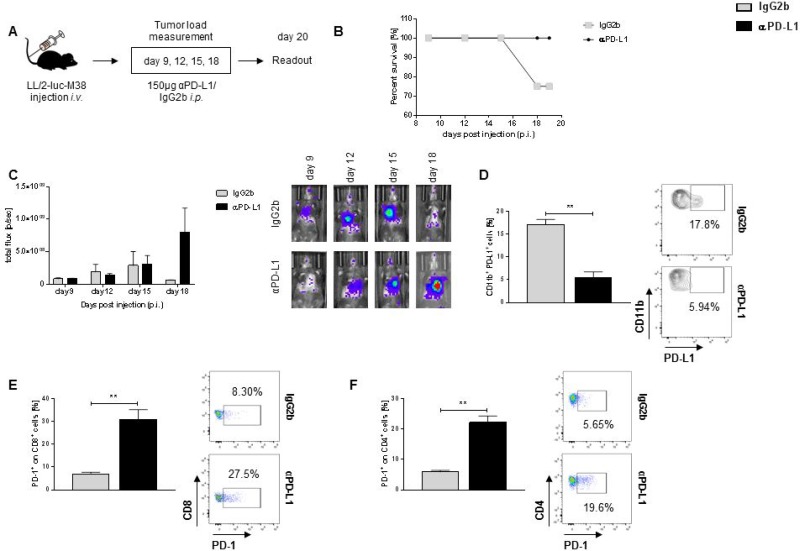
αPD-L1 antibody therapy resulted in better survival rates but is also associated with increase expression of PD-1 in TIL (**A**) Experimental design. Mice were injected *i.v.* with 1 × 10^6^ LL/2-luc-M38 cells. Starting at day 9 post injection, every three days mice were treated *i.p.* with 150μg αPD-L1 or related isotype control IgG2b. Lung tumor development was detected via bioluminescence. (**B**) αPD-L1 antibody treatment of naive tumor bearing mice resulted in a better survival rate compared to tumor bearing mice treated with related isotype control IgG2b (n_IgG2b_ = 4; n_αPD-L1_ = 4). (**C**) αPD-L1 antibody treatment did not resulted in an altered lung tumor development compared to mice treated with IgG2b (n_IgG2b_ = 4; n_αPD-L1_ = 4). (**D**) αPD-L1 antibody treatment successfully inhibited the PD-L1 expression on CD11b^+^ tumor infiltrating cells (n_IgG2b_ = 3; n_αPD-L1_ = 4). (**E**) Blockade of PD-L1 is associated with an increased infiltration of PD-1^+^ CD8^+^ T cells in the lungs of tumor bearing mice compared to tumor bearing mice treated with IgG2b (n_IgG2b_ = 3; n_αPD-L1_ = 4). (**F**) αPD-L1 treatment resulted in a significantly enhanced accumulation of PD-1^+^ T CD4^+^ cells in the lungs of tumor bearing compared to mice treated with related isotype control IgG2b (n_IgG2b_ = 3; n_αPD-L1_ = 4). Data are presented as mean values ± SEM; unpaired *t*-test ^*^p < 0.05, ^**^p < 0.01, ^***^p < 0.001.

As it has been reported that PD-L1 expression in tumor patients is linked to a better response to immunotherapy [13] and we found upregulated *PDL1* expression in the TU region of our patient cohort, we next examined the effect of *in vivo* PD-L1 blockade in our murine model of lung cancer. Mice were intraperitoneally treated with αPD-L1 antibody or IgG2b isotype control at described days and tumor development was measured via *in vivo* bioluminescence (Figure 2A, 2C). Tumor bearing mice *in vivo* treated with αPD-L1 antibodies show a better survival outcome compared to tumor bearing mice *in vivo* treated with the IgG2b isotype control (Figure 2B). Despite the better survival rate, we could neither detect an altered tumor development in both groups (Figure 2C) nor a change in weight loss during the tumor development (Supplementary Figure 2A). To evaluate whether *in vivo* blockade of PD-L1 was successful, we analysed PD-L1 expression on total lung cells derived from tumor bearing mice. PD-L1 expression was successfully reduced during *in vivo* blockade on CD11b^+^ cells (Figure 2D) as well as on total cells (Supplementary Figure 2B) and on epithelial cells, characterized as EpCAM^+^ cells (Supplementary Figure 2C). Since PD-1 is the binding partner to PD-L1 [22], we next analysed the expression of PD-1 on tumor infiltrating cells. Notably, we found an upregulation of PD-1 on CD8^+^ lung tumor infiltrating T cells (Figure 2E) as well as on CD4^+^ lung tumor infiltrating T cells in mice *in vivo* treated with αPD-L1 antibody compared to isotype control IgG2b (Figure 2F).

### Targeted deletion of host STAT1 results in induced lung tumor development

**Figure 3:**
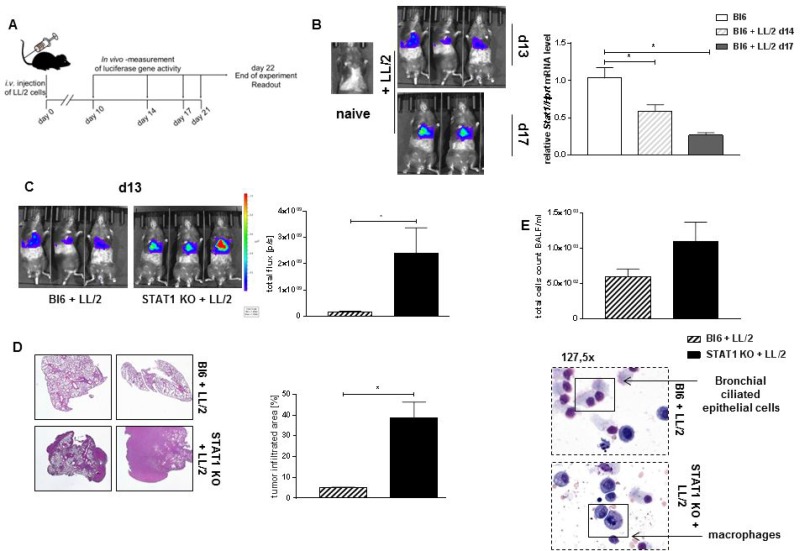
STAT1 deficiency is associated with induced tumor development (**A**) Experimental design for the induction of experimental lung adenocarcinoma. Mice were injected with 1 × 10^6^ LL/2-luc-M38 cells (LL/2 cells) intravenously (i.v.). Tumor development was measured with the help of an *in vivo* bioluminescence imaging system at described days. Experiment ended at day 14 to day 20. (**B**) *Stat1* mRNA was measured in total lung cells of tumor bearing Bl6/C67j (Bl6) mice at day 13 and day 17 after lung tumor induction. *Stat1* mRNA level decreases during the progression of lung adenocarcinoma in total lung cells (n_Bl6_ = 4; n_Bl6 + LL/2 d14_ = 5; n_Bl6+ LL/2 d17_ = 2). (**C**) Loss of STAT1 leads to a significantly increased tumor development compared to tumor bearing wild-type mice (n_Bl6 + LL/2_ = 10; n_STAT1 KO + LL/2_ = 9). Both groups showed comparable disease incidence and no mortality. Two of four representative experiments are shown. (**D**) Hematoxylin and Eosin (H&E) showed increased tumor infiltrated area in STAT1 knockout (STAT1 KO) mice compared to tumor bearing Bl6 mice (_nBl6 + LL/2_ = 3; _nSTAT1 KO + LL/2_ = 3). (**E**) No altered total cell count in the bronchial lavage fluid (BALF) between tumor bearing STAT1 KO mice and Bl6 mice was detected. May-Gruenwald-Giemsa staining demonstrated that the BALF of tumor bearing STAT1 KO mice is characterized by a strong infiltration of immune cells, especially by monocytes and macrophages (n_Bl6 + LL/2_ = 5; _nSTAT1 KO + LL/2_ = 5). Data are presented as mean values ± SEM; unpaired *t*-test ^*^p < 0.05, ^**^p < 0.01, ^***^p < 0.001.

Since responders to *in vivo* blockade of PD-1 show a trend to upregulation of *Stat1* expression in total lung cells, we next examined the role of STAT1 during lung adenocarcinoma development. To induce lung tumor development Bl6/C57j mice were injected with LL/2-luc-M38 cells intravenously, tumor load was measured at described days via *in vivo* bioluminescence (Figure 3A–3C). Total cells from tumor bearing lungs from Bl6/C57j mice were isolated at day 13 and 17 after tumor induction. *Stat1* mRNA level decreased with progressed lung tumor development in total lung cells derived from tumor bearing mice (Figure 3B). Because of this finding, we next examined the total deletion of host STAT1 on lung tumor development. Therefore, we used STAT1 knockout (KO) mice in our murine model of lung adenocarcinoma. STAT1 KO mice show a rapid and significantly severe lung tumor development compared to tumor bearing Bl6/C67j mice (Figure 3C). Hematoxylin and Eosin (H&E) staining verified findings of *in vivo* bioluminescence and showed an increased tumor infiltrated area in lungs of STAT1 KO mice compared to Bl6/C57j mice (Figure 3D). Bronchoalveolar lavage fluid (BALF) was taken at the end of experiment. Total cell count in the BALF was not altered between tumor bearing STAT1 KO mice and Bl6/C57j mice. However, cells in the BALF were predominantly characterized as macrophages via May-Grünwald-Giemsa staining in STAT1 KO mice, whereas in tumor bearing Bl6/C56j wild type mice predominantly bronchial ciliated epithelial cells were found (Figure 3E).

### Loss of STAT1 impairs differentiation into M1-like macrophages

Since we found a strong accumulation of PD-L1^+^ macrophages in the BALF of tumor bearing STAT1 KO mice, we next wanted to examine the effect of STAT1 on the differentiation of macrophages. Macrophages derived from the bone marrow are recruited into different tissues to react to different cytokines or immune stimuli [23] therefore we analysed bone marrow derived macrophages from naïve STAT1 KO mice in order to investigate the effect of STAT1 on differentiation of macrophages. Bone marrow cells were isolated from fibula and tibia. Cells were cultured with GM-CSF for ten days and differentiated into M1-like macrophages with the respective cytokine mixture for 48 h (Supplementary Figure 3A). Naïve bone marrow derived M1 macrophages differentiated from STAT1 KO mice show a strong defect in *Nos2* mRNA expression (Supplementary Figure 3B). Supernatants collected after differentiation into M1 macrophages have lower levels of soluble TNFα compared to M1-like macrophages derived from Bl6/C57j mice (Supplementary Figure 3C). Interestingly, we found significantly upregulated mRNA expression of *Pd1* in Stat1 deficient M1 macrophages compared to wild-type M1 macrophages (Supplementary Figure 3D). As macrophages are able to directly eliminate tumor cells [4] by secreting inflammatory cytokines, we next evaluated the ability of Stat1 deficient M1 macrophages to kill LL/2-luc-M38 lung adenocarcinoma cells used to induce lung tumor in mice. For this assay LL/2-luc-M38 were seeded and incubated with supernatants collected from M1 differentiated macrophages diluted in DMEM medium (conditioned medium = CM). After 24 h incubation with CM, cell viability was measured via bioluminescence (Supplementary Figure 3E). LL/2-luc-M38 cultured with 20% or 50% of CM from STAT1-/- mice showed significantly increased cell viability compared to LL/2-luc-M38 cells that were incubated with CM derived from wild-type M1-like macrophages (Supplementary Figure 3F).

### STAT1 deficiency is associated with accumulation of PD-L1^+^ macrophages in tumor bearing mice

**Figure 4:**
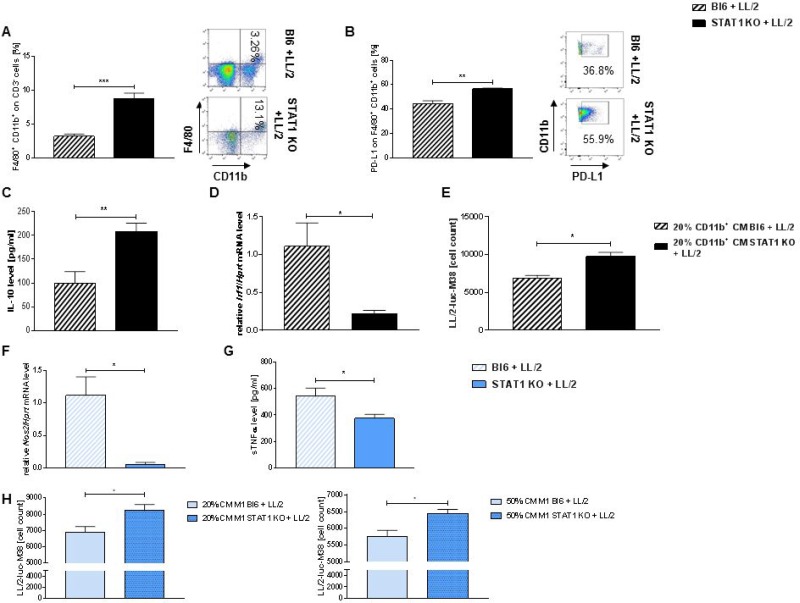
Loss of STAT1 is associated with increased infiltration of tumor associated macrophages and PD-L1 overexpression (**A**) Accumulation of tumor infiltrating macrophages characterized as CD11b^+^ F4/80^+^ previously gated on CD3^−^ cells in tumor bearing Stat1 deficient mice compared to tumor bearing WT mice (n_Bl6 + LL/2_ = 11; n_STAT1 KO + LL/2_ = 11). (**B**) Loss of Stat1 leads to an overexpression of PD-L1 on lung tumor infiltrating macrophages (CD11b^+^ F4/80^+^) during lung adenocarcinoma development compared to wild-type controls (n_Bl6 + LL/2_ = 11; n_STAT1 KO + LL/2_ = 11). (**C**) Significantly enhanced secretion of IL-10 from total lung cells was measured in the supernatants of tumor bearing STAT1 KO mice compared to Bl6 mice (n_Bl6 + LL/2_ = 9; n_STAT1 KO + LL/2_ = 9). (**D**) Loss of Stat1 leads to a significantly decreased expression of *Irf1* in total lung cells of tumor bearing mice compared to Bl6 controls (n_Bl6 + LL/2_ = 4; n_STAT1 KO + LL/2_ = 5). (**E**) Conditioned medium (CM) from CD11b^+^ tumor infiltrating macrophages from Stat1 deficient mice failed to reduce cell viability of LL/2-luc-M38 cells compared to CD11b^+^ derived from tumor bearing Bl6 mice (n_Bl6 + LL/2_ = 3; n_STAT1 KO + LL/2_ = 3). (**F**) Loss of Stat1 leads to a defect of *Nos2* expression on M1 like bone marrow derived macrophages in tumor bearing mice (n_Bl6 + LL/2_ = 10; n_STAT1 KO + LL/2_ = 6). (**G**) M1-like bone marrow derived macrophages derived from tumor bearing STAT1 KO secreted significant lower levels of soluble TNFα compared to M1 macrophages derived from tumor bearing Bl6 mice (n_Bl6 + LL/2_ = 7; n_STAT1 KO + LL/2_ = 7). (**H**) Enhanced LL/2-luc-M38 cell viability was detected when lung adenocarcinoma cells were incubated with CM of M1 macrophages derived from tumor bearing STAT1 KO mice compared to tumor bearing wild-type mice (n_Bl6 + LL/2_ = 10; n_STAT1 KO + LL/2_ = 6). Data are presented as mean values ± SEM; unpaired *t*-test ^*^p < 0.05, ^**^p < 0.01, ^***^p < 0.001.

As we could observe that the loss of STAT1 leads to a defect in M1 macrophages in the bone marrow in naïve mice, we next wanted to investigate the consequences of this phenomenon in our murine model of lung adenocarcinoma model. We first found a strong accumulation of CD11b^+^ F4/80^+^ cells, which were previously gated on CD3^−^ cells in the lungs of tumor bearing STAT1 KO mice compared to tumor bearing wild-type control (Figure 4A). Here we found that these lung tumor infiltrating macrophages (CD11b^+^ F4/80^+^ gated on CD3^−^) strongly expressed PD-L1 in tumor bearing STAT1 KO mice compared to tumor bearing Bl6/C67j mice (Figure 4B). Supernatants from total lung cells derived from tumor bearing mice showed that an increased secretion of IL-10 in STAT1 KO mice compared to wild-type controls (Figure 4C). As we found a defect in *Nos2* expression in Stat1 deficient M1 macrophages (Supplementary Figure 3B), we next wanted to analyze Interferon Regulatory Factor 1 (IRF1). IRF1 is described to be able to directly induce NO synthase [24] and is therefore an important factor to eliminate tumor cells. Indeed, we found a strong reduction of *Irf1* mRNA level in total lung cells derived from tumor bearing STAT1 KO mice compared to tumor bearing wild-type controls (Figure 4D). In order to verify that the upregulation of PD-L1 on Stat1 deficient macrophages, increased IL-10 secretion and impaired *Irf1* expression also impairs the killing function of Stat1 deficient macrophages, we next isolated CD11b^+^ cells from tumor bearing lungs. Purity of isolated CD11b^+^ was analysed via flow cytometry and a purity of around 90% was detected (Supplementary Figure 4A). LL/2-luc-M38 cells were incubated with conditioned medium (CM) derived from isolated CD11b culture. CM was diluted in DMEM medium. LL/2-luc-M38 cells were incubated with a 20% dilution of CM derived from CD11b culture. In line with our previous results, cell viability of LL/2-luc-M38 cells was higher when incubated with CM derived from tumor bearing Stat1 deficient CD11b^+^ cells (Figure 4E). Cell count was determined via a standard curve (Supplementary Figure 4C). To strengthen our results, we repeated differentiation assay of M1 macrophages but in tumor bearing mice. Similar to our results in naïve mice, we found a strong downregulation of *Nos2* expression in Stat1 deficient M1 macrophages derived from tumor bearing mice (Figure 4F) as well as decreased secretion of soluble TNFα in the supernatant derived from M1 macrophages differentiated from tumor bearing STAT1 KO mice (Figure 4G). In accordance to these findings LL/2-luc-M38 cell viability assay showed an increased survival of murine tumor cells, when incubated with CM derived from Stat1 deficient M1 macrophages (Figure 4H).

### Impaired activation of cytotoxic T cells infiltrating the tumor of Stat1 deficient mice

**Figure 5:**
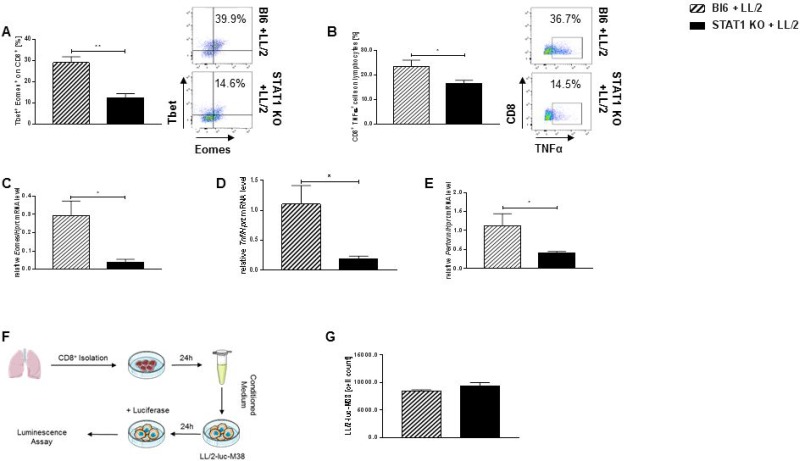
Loss of Stat1 negatively impairs cytotoxic capacity of tumor infiltrating CD8^+^ T lymphocytes (**A**) Dramatic reduction of Tbet^+^ Eomes^+^ previously gated on CD8^+^ cytotoxic lymphocytes in the lungs of tumor bearing STAT1 KO mice compared to CD8^+^ tumor infiltrating T lymphocytes in tumor bearing Bl6 mice (n_Bl6 + LL/2_ = 6; n_STAT1 KO + LL/2_ = 5). (**B**) Stat1 deficiency resulted in a decreased capability of lung CD8^+^ T lymphocytes to produce TNFα during lung tumor development compared to tumor bearing wild-type controls (n_Bl6 + LL/2_ = 6; n_STAT1 KO + LL/2_ = 6). (**C**) *Eomes* mRNA level were significantly reduced in total lung cells from Stat1 deficient mice compared to tumor bearing Bl6 mice (n_Bl6 + LL/2_ = 4; n_STAT1 KO + LL/2_ = 5). (**D**) Loss of Stat1 leads to a reduced mRNA expression of *Tnf* in total lungs cells compared to total lung cells derived from tumor bearing wild-type mice (n_Bl6 + LL/2_ = 4; n_STAT1 KO + LL/2_ = 5). (**E**) Stat1 deficiency leads to a downregulation of *Perforin* mRNA expression in total lung cells derived from tumor bearing mice (n_Bl6 + LL/2_ = 4; n_STAT1 KO + LL/2_ = 5). (**F**) Experimental design of an *in vitro* bioluminescence cytotoxic assay. CD8^+^ T cells were purified from tumor bearing lungs of Bl6 and STAT1 KO mice. Supernatants from CD8^+^ T cells were diluted with DMEM (conditioned medium = CM). LL/2-luc-M38 cells were cultured for 24 h with CM. Cell viability was detected via bioluminescence. (**G**) No alteration in the cell viability of LL/2-luc-M28 lung adenocarcinoma cells co-cultured with CM from CD8^+^ tumor infiltrating T lymphocytes between STAT1 KO and Bl6 mice (n_Bl6 + LL/2_ = 3; n_STAT1 KO + LL/2_ = 3). Data are presented as mean values ± SEM; unpaired *t*-test ^*^p < 0.05, ^**^p < 0.01, ^***^p < 0.001.

Macrophages are able to modulate antigen cross-presentation and T cell activation. Previous studies demonstrated that macrophages are able to activate CD8^+^ T cells for proliferation and T cells cytokine production [25]. Since we could clearly demonstrate a defect in the killing capacity of Stat1 deficient macrophages, we next wanted to investigate whether this defect also has consequences for T cell activation. Indeed, we found a reduced number of Tbet^+^ Eomes^+^ cells previously gated on CD8^+^ cells in tumor bearing lungs from STAT1 KO mice (Figure 5A). Further on, we could demonstrate that tumor bearing lungs from STAT1 KO mice are significantly less infiltrated by CD8^+^ TNFα^+^ T cells compared to tumor bearing Bl6/C57j mice (Figure 5B). mRNA analysis of total lung cells derived from tumor bearing mice showed that STAT1 KO mice have a strong downregulation of *Eomes* (Figure 5C), *Tnf* (Figure 5D) and *Perforin* (Figure 5E) compared to total lung cells derived from tumor bearing wild-type mice. In order to analyze whether the observed effect is based on impaired activation of CD8^+^ T cells because of impaired macrophage function or if Stat1 deficiency leads to impaired CD8+ T cells activation we isolated CD8^+^ T cells from tumor bearing STAT1 KO mice. CM derived from CD8+ T cell culture was diluted in DMEM medium and LL/2-luc-M38 cells were cultured with 20%CM and cell viability was analyzed after 24 h (Figure 5F, Supplementary Figure 5A). No difference was detected in cell viability of LL/2-luc-M38 cells between incubation with CM obtained from Stat1 deficient CD8^+^ T cells and CM derived from CD8^+^ T cells from Bl6/C57j mice (Figure 5G). As positive control CM from CTLL2 cells was used (Supplementary Figure 5C). Purity of isolated CD8^+^ T cells from tumor bearing lungs was similar between both mice strains (Supplementary Figure 4B). Cell count was determined via a standard curve (Supplementary Figure 5B). In order to strengthen our hypothesis that Stat1 deficiency had no impact on CD8^+^ T cells but Stat1 deficient macrophages, were not able to recruit CD8^+^ cells in the tumor microenvironment we also isolated lung CD8^+^ cells from naïve STAT1 KO mice and Bl6/C57j mice. CM from naïve lung CD8^+^ T cells from STAT1 KO mice or Bl6/C57j mice was diluted in to 20% or 50% CM. No difference in cell viability of murine lung cancer was detected when LL/2-luc-M38 cells were incubated with either CM from naïve lung CD8^+^ T cells from STAT1 KO or wild-type mice (Supplementary Figure 5D). Cell vitality was determined via standard curve (Supplementary Figure 5B). We further on, could demonstrate that there is no difference in apoptosis between isolated lung CD8^+^ T cells derived from naïve STAT1 KO mice or naïve wild-type mice (Supplementary Figure 5E).

### Stat1 deficiency leads to an upregulation of suppressive T cells in the tumor microenvironment

**Figure 6:**
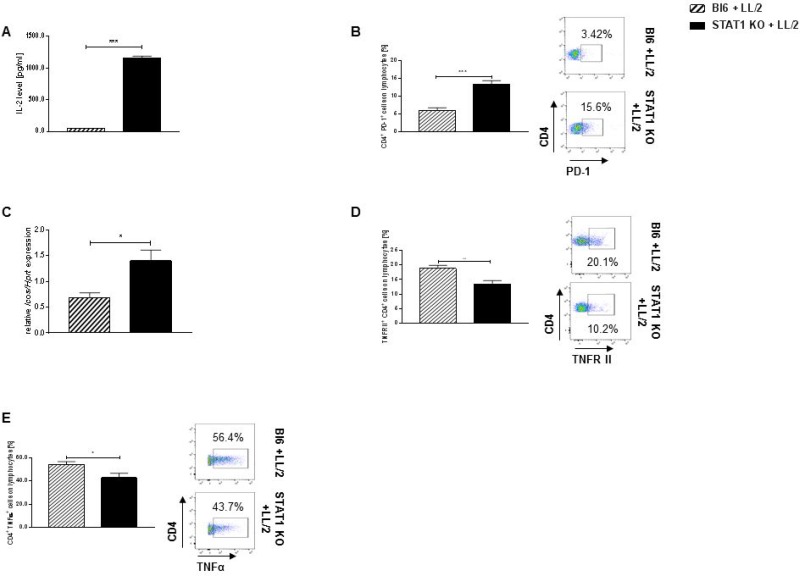
Stat1 deficiency negatively impacts cytotoxic CD4^+^ tumor infiltrating T lymphocytes (**A**) Total lung cells isolated from tumor bearing STAT1 KO mice secreted higher levels of IL-2 compared to total lung cells derived from tumor bearing Bl6 mice (n_Bl6 + LL/2_ = 5; n_STAT1 KO + LL/2_ = 5). (**B**) CD4^+^ tumor infiltrating lymphocytes strongly upregulated PD-1 in tumor bearing STAT1 KO mice compared to tumor bearing controls (n_Bl6 + LL/2_ = 5; n_STAT1 KO + LL/2_ = 4). (**C**) Stat1 deficiency leads to an increase of *Icos* in mRNA derived from total lung cells of tumor bearing mice compared to tumor bearing Bl6 mice (n_Bl6 + LL/2_ = 4; n_STAT1 KO + LL/2_ = 4). (**D**) Loss of Stat1 leads to a defect of TNFα production in CD4^+^ tumor infiltrating lymphocytes (n_Bl6 + LL/2_ = 6; n_STAT1 KO + LL/2_ = 6). (**E**) Tumor infiltrating CD4^+^ T lymphocytes of STAT1 KO mice express lower numbers of the TNFR II compared to tumor bearing wild-type controls (n_Bl6 + LL/2_ = 6; n_STAT1 KO + LL/2_ = 6). Data are presented as mean values ± SEM; unpaired-test ^*^p < 0.05, ^**^p < 0.01, ^***^p < 0.001.

As we found a significant upregulation of PD-L1 in the lung tumor microenvironment of STAT1 KO mice, we next asked about the influence of STAT1 on PD-1. PD-1 is the main binding partner to PD-L1 [22]. It has been previously reported that the PD-1/PDL1 signaling pathway promotes the proliferation, maintenance and suppressive function of T regulatory cells (Tregs) located in the tumor microenvironment [26]. Therefore, we next started to analyse regulatory T cells in tumor bearing STAT1 KO mice. First, we found a significantly increased secretion of IL-2 from total lung cells derived from tumor bearing STAT1 KO mice compared to tumor bearing Bl6/C57j mice (Figure 6A). Interestingly, we could not find an alteration in Foxp3^+^ CD4^+^ previously gated on CD4^+^ CD25^+^ tumor infiltrating T cells between both mice strains (Supplementary Figure 6A) but an enhanced accumulation of CD4^+^ PD-1^+^ T cells in the lung tumors of STAT1 KO mice (Figure 6B). Moreover, we found a significantly increased mRNA expression of the suppressive surface marker CD278 (*Icos*) in total lung cells derived from tumor bearing STAT1 KO mice compared to tumor bearing wild-type mice (Figure 6C) but no alteration was found on *Ctla4* mRNA expression between both mice strains (Supplementary Figure 6B). Since we found a strong upregulation of suppressive markers, we next asked whether cytotoxic function was affected by Stat1 deficiency. We first found that, tumor infiltrating CD4+ T cells show no difference in the expression of the TNFR I (Supplementary Figure 6C) but a downregulation of the TNFR II was detected on CD4^+^ T cells in the lungs of tumor bearing STAT1 KO mice compared to tumor bearing Bl6/C57j mice (Figure 6D). In line with this finding, we found a reduced production of TNFα by tumor infiltrating Stat1 deficient CD4^+^ cells (Figure 6E).

In conclusion, these results indicate that the loss of STAT1 leads to an upregulation of PD-L1, especially on tumor infiltrating macrophages, which is probably linked to a more suppressive tumor microenvironment characterized by CD4+PD1+T cells and a dysfunctional recruitment of cytotoxic CD8^+^ T cells in the tumor. Further on, reduced TNFα secretion seems to be associated with the inability of tumor infiltrating lymphocytes and macrophages to directly kill the LL/2-luc-M38 tumor cells.

## DISCUSSION

One of the major limitation in the use of ICB therapies is the low percentage of patients that successfully respond to ICB [10–12]. Beside the problem that patients do not benefit from treatment resistance mechanisms are defined as adaptive immune resistance and acquired immune resistance. One documented case is the constitutive expression of PD-L1 that may inhibit anti-tumoural T cell responses. However, it is controversially discussed whether constitutive expression of PD-L1 is associate with increased or decreased likelihood to respond to immunotherapy [27]. In our murine model of lung adenocarcinoma *in vivo* blockade of PD-L1 resulted in an upregulation of PD-1 in tumour infiltrating T lymphocytes. PD-1 is known to induce T cell exhaustion which further on negatively influences the activation state and cytotoxic properties of cytotoxic T lymphocytes [28]. In line with this we found increased infiltration of PD-1^+^ T cells in the tumor microenvironment and reduced production of TNFα by CD4^+^ and CD8^+^ T cells in the lung of STAT1−/− mice bearing tumour. TNFα has been previously described as an important anti-tumoural molecule to induce apoptosis in our murine lung adenocarcinoma cell line LL/2-luc-M38 [19]. Reduced TNFα secretion is also linked to the inability to suppress tumor cell proliferation and to induce tumor progression. Further on, reduced TNFα is described to lead to impaired recruitment of lymphocytes in the tumor [29] and therefore is associated with enhanced tumor development. In general, TNFα is a pleiotropic cytokine, characterized with antitumoural and protumoural functions [5]. It exists in two isoforms, a transmembrane bound TNFα and a soluble form [30]. Both isoforms, soluble and transmembrane bind to the TNF receptor (TNFR) I and TNFR II [31, 32]. In our murine lung cancer model, we found a significantly reduced secretion of soluble TNFα by M1 macrophages and impaired secretion of soluble TNFα by CD4^+^ and CD8^+^ T cells. Of note, the transmembrane isoform of TNFα is also able to kill tumor cells [33]. Since we found a downregulation of the TNFR II but an unaltered expression of TNFR I in tumor bearing STAT1 KO mice, it is notably that the transmembrane form of TNFα is able to exerts its cytotoxicity via both receptors. However, previous reports show that STAT1 mediates the transmembrane TNFα induced formation of death inducing signaling complex and apoptosis via the TNFR I [34]. Taken together the loss of Stat1 leads to an impaired TNFα mediated immune signaling, resulting in a complete defect to kill tumor cells in our murine model of lung adenocarcinoma. Previous studies in melanoma patients used TNFα antibodies to overcome the resistance to anti-PD-1 therapy [35]. Our results showed that in NSCLC the blockade of TNFα would favor the tumor growth and probably would show a completely different effect as in melanoma treatment and which further on demonstrated the dual effects of TNFα in different cancer types. Further experiments in this direction are needed. Moreover, we could demonstrate that a successful blockade of PD-1 is associated with a trend to increased *Stat1* expression in total lung cells derived from tumor bearing mice. Additionally, we demonstrated in our murine lung cancer model and our human patient cohort that increased expression of *STAT1* is associated with decreased tumor size. In our human patient cohort, we found this inverse correlation especially for the *STAT1β* isoform. STAT1α and STAT1β are both able to accumulate on the IFNγ-induced IRF-1 promotor, which was demonstrated by *in vivo* chromatin immunoprecipitation but one of the main differences is that only STAT1α is capable to recruit CBP (CREB binding protein)/p300 and to stimulate transcription [36]. The function of CBP/p300 is highly discussed. These proteins appear to contribute to certain tumor-suppressor pathways but both proteins are also essential for the actions of many oncogenes [37]. As STAT1α only correlates inversely with the tumor size in the TU region of NSCLC patients, this phenomenon might be explained by controversial function of the CBP/p300 proteins, recruited by STAT1α in the CTR and TU from patients suffered from NSCLC, probably depending on the different microenvironment. Another important aspect for STAT1β being the more successful key marker in tumor defense than STAT1α is the fact that STAT1β acts as a dominant negative regulator in IFNγ signaling [38] and that the best characterized signaling axis for PD-L1 is its induction mediated by IFNγ [1].

All these results indicate STAT1 as a potential new key target for successful combinatory ICB.

## MATERIALS AND METHODS

### Human subjects and study population

This study was performed at the Friedrich-Alexander-Universität Erlangen-Nürnberg in Geramany and was approved by the ethics review board of the Friedrich-Alexander-Universität Erlangen-Nürnberg (Re-No: 56_12B; DRKS-ID: DRKS00005376). Sixty-five patients that suffered from NSCLC underwent surgery and gave their approval to be enrolled in this study in an informed written consent. The patients were conducted in accordance with the ethical guidelines of the Declaration of Helsinki. The diagnosis of lung cancer was based on pathological confirmation. The histological classification of lung cancers was performed in accordance to the WHO (World Health Organization), formulated in 2004. TNM classification of patients that suffered from NSCLC was performed in accordance to the International Association for the Study of Lung Cancer (IASLC) in 2010. During lung surgery, three regions were taken: the tumoural region (TU: solid tumor tissue), the peri-tumoural region (PT: up to 2 cm away from the solid tumor) and a tumor free control regin (CTR: at least 5 cm away from the solid tumor). This cohort of NSCLC patients was previously derived in [16].

### Protein extraction and Western blot analysis

For protein extraction, lung tissue samples were lysed in RIPA buffer (Thermo Fisher Scientific, Waltham, MA, USA) added with an inhibitor cocktail (Roche Diagnostics, Mannheim, Germany). Lung tissue samples were homogenized using SpeedMill PLUS (Analytik Jena, Jena, Germany) and innuSPEED lysis Tube P (Analytik Jena). After homogenization samples were centrifuged (5 min, 3000 rpm, 4°C), supernatants were collected and incubated on ice for 45 min followed by centrifugation (5 min, 3000 rpm, 4°C; 45 min, full speed, 4°C). Protein concentration was determined using Bradford Assay (Protein Assay Dye Reagent Concentration, Bio-Rad, Munich, Germany). Western blot analysis to detect Phospho-STAT1 Tyr701 (1/1000, cell signaling, Danvers, MA, USA), PD-L1 E1L3N (1/1000, cell signaling) and b-Actin (1/500, sc-1616, Santa Cruz, TX, USA) was performed as described in [39] with 50 μg of total protein. Quantification of total p-Tyr-STAT1 and PD-L1 was performed using AlphaView Software for FluorChem Systems (Biozym Scientific, Oldendorf, Germany).

### Cell lines

The murine lung adenocarcinoma cell line LL/2-luc-M38 was purchased and authenticated from Caliper LifeScience (Bioware cell line, Caliper LifeScience, Waltham, MA, USA). All Caliper LifeScience cell lines are confirmed to be pathogen-free by IMPACT profile I (PCR) at the University of Missouri Research Animal Diagnostic and Investigation Laboratory. Luciferase expression is coupled to a neomycin-resistance gene which renders the cell resistant to geneticin (G418). In order to select only luciferase expressing tumor cells, LL/2-luc-M38 cells were treated periodically with G418 solution (500 μg/ml, Sigma-Aldrich, Taufkirchen, Germany). Three to four cell passages between thawing and usage were performed before cells were injected into the tail vein. LL/2-luc-M38 cells were cultured in DMEM (Thermo Fisher Scientific) supplemented with 10% FCS, 1% Pen/Strep and 1% l-Glut at 37°C and 5% CO_2_. Mycoplasma contamination was detected using Mycoplasma Detection Kit (Absource Diagnostics GmbH) according to the manufacturer’s instructions (latest test: August, 9th, 2016).

The murine CTLL2 cell line was kindly provided to use by PD Dr. Ulrike Schleicher from the Institute of Microbiology, Universitätsklinikum Erlangen-Nürnberg. Cells were cultured in RPMI-1640 medium (Gibco, Thermo Fisher Scientific) supplemented with 10% FCS, 1% Pen-Strep, 1% L-Glut and 4 ng/ml IL-2 (PeproTech, GmbH, Hamburg, Germany) at 37°C and 5% CO_2_.

### RNA isolation and cDNA synthesis

Human lung tissues were homogenized with the usage of Precellys Lysing Kits (Bertin Technologies, Montigny-le-Bretonneux, France) using the benchtop homogenizer Minilys (Bertin Technologies) as written in the manufacturer’s instructions. Total RNA derived from human samples and murine cells was isolated using peqGold RNA Pure (Peqlab, Erlangen, Germany) in accordance to the manufacturer’s protocol. RNA was reverse-transcribed into cDNA using RevertAid™ First Strand cDNA Synthesis Kit (Fermentas, St. Leon-Rot, Germany) in accordance to the manufacturer’s protocol.

### Quantitative real-time PCR (qPCR)

qPCR was performed with cDNA using iTaq Universal SYBR Green Supermix (BioRad, Munich, Germany) in a total volume of 20 μl. qPCR primers were designed and purchased at Eurofins-MWG-Operon, Ebersberg, Germany. Reactions (50 cycles, initial activation at 98°C, 2min, denaturation at 95°C, 5 min, hybridization at 60°C, 10 min) were performed using the CFX-96 Real-time PCR Detection System (BioRad) and analysed by the CFX Manager Software (BioRad). Relative quantification was performed using the 2-ΔΔCT method, Hprt (hypoxanthine-guanine-phosphoribosyltransferase was used as a housekeeping gene. The following sequences were used for human qPCR analysis: *HPRT* for 5′-TGACACTGGCAAAACAATGCA-3′ rev 5′-GGTCCTTTTCACCAGCAAGCT-3′; *PDL1* for 5′-AGCAAAGTGATACACATTTTGGAG-3′ rev 5′-CC CCGATGAACCCCTAAACC-3′, *STAT1α* for 5′-CAC CAGAGCCAATGGAACTT-3′ rev 5′-ACAGAGCCCA CTATCCGAGA-3′, *STAT1β* for 5′-CTTTCCCTGACAT CATTCGCA-3′ rev 5′-AAGGCTGGCTTGAGGTTTGT A-3′. For murine qPCR analysis the following primers were used: *Hprt*: for 5′-GCCCCAAAAT GGTTAAGGTT-3′ rev 5′-TTGCGCTCATCTTAGGCT TT-3′; *Nos2*: for 5′-AGACCTCAACAGAGCCCTCA-3′ rev 5′-TCGAAGGTGAGCTGAACGAG-3′; *Tnf*: for 5′-AGCCCCCAGTCTGTATCCTT-3′ rev 5′-CTCCCT TTGCAGAACTCAGG-3′; *Perforin* for 5′-GATGTGAACCCTAGGCCAGA-3′ rev 5′-GGTTTTTGTACCAGGCGAAA-3′; *Tbet* for 5′-TTCCCATTCCTGTCCTT CACCG-3′ rev 5′-GGAAGGTCGGGGTAAAAAC-3′; *Ctla4* for 5′-GGATCCTTGTCGCAGTTAGC-3′ rev 5′-TCACATTCTGGCTCTGTTGG-3′; *Icos* for 5′-GTGC AGCTTTCGTTGTGGTA-3′ rev 5′-TCAGGGGAACTA GTCCATGC-3′; *Stat1* for 5′-CATGGCTGCCGAGAACA TAC-3′ rev 5′-TCTGGTGCTTCCTTTGGTCT −3′; *Stat2* for 5′-TCAGACTTACCAGGCTTCCG-3′rev 5′-GTCAAGAAGCCGAAGTCCCA-3′; *Stat3* for 5′-GCT TCC TGC AAG AGT CGA AT-3′ rev 5′-ATT GGC TTC TCA AGA TAC CTG-3′.

### Luminescence/cell viability assay

7 × 10^^3^ LL/2-luc-M38 cells were seeded in a 96 well plate and incubated for 24 h at 37°C, 5% CO_2_ in DMEM supplemented with 10% FCS, 1% L-Glut and 1% Pen/Strep. After 24h medium was removed and LL/2-luc-M38 cells were washed with PBS and incubated with 20% or 50% conditioned medium (CM) derived from M1 macrophage culture or CD8^+^ cell culture diluted in DMEM plus supplements. After another 24 h incubation at 37°C, 5% CO_2_, conditioned medium was removed and LL/2-luc-M38 were incubated with a 15 μg/ml luciferin (Promega, Mannheim, Germany) solution (diluted in PBS) to detect luminescence intensity using Centro XS^3^ LP 960 Mircoplate Luminometer (Berthold Technologies, Bad Widlbach, Germany). Detected luminescence is proportional to the living cell number. Cell viability is therefore calculated via standard curve.

### ELISA

Enzyme-linked immunosorbent assay (ELISA) was used to detect the cytokine concentration in cell culture supernatants. ELISA was performed in accordance to the manufacturer’s protocol. Murine IL-2, IL-10 and TNFα ELISA Duo Sets were purchased from BD BioScience.

### Mice

STAT1 knockout (KO) mice were kindly donated by Dr. Chiriac from the Department Medicine 1, Universitätsklinikum Erlangen-Nürnberg. STAT1 KO mice are on Bl6/C57j background. Bl6/C57j mice and STAT1 KO mice were bred in house under a 12h light/dark cycle at the local animal care facility of the Friedrich-Alexander-Universität Erlangen-Nürnberg, Hartmannstraße 14, 91052 Erlangen. Food and water were provided *ad libitum*. All animal procedures were approved by the respective government in accordance to the German animal protection law and carried out by skilled experimenters (Az 55.2–2532.1-36/13).

### Murine model of lung adenocarcinoma and *in vivo* imaging

LL/2-luc-M38 cells were cultured in DMEM supplemented with 10% FCS, 1% L-Glut and 1% Pen/Strep. 1 × 10^^6^ LL/2-luc-M38 in a total volume of 200 μl DMEM without any supplements were injected intravenously into the tail vein of 6–8 weeks old, female mice. At described time points, mice were shaved and weighted and luciferin (0.15 mg/g body weight; Promega) was injected intraperitoneally. After 20min incubation, mice were anaesthetized with isoflurane and luciferase activity was measured by the IVIS Spectrum *In Vivo* Imaging System (PerkinElmer, Waltham, USA). Luciferase activity was measured by detecting the emitted photons per second (intensity). Lung tumor load analysis was done as previously described in [40]. *In vivo* blocking of PD-1, PD-L1 or the respective isotype controls IgG2a, IgG2b was done by intraperitoneal injection. αPD-1 (RMP1-14, Hölzel Diagnostika) and rat IgG2a 2A3 isotype control or αPDL1 (B7-H1, Hölzel Diagnostika) and rat IgG2b κ were injected at a concentration of 150 μg/mouse every 4 days for maximal four injections starting at day 8 after tumor induction. At day 20 post induction of the LL/2-luc-M38 cells, total lung cells were isolated as previously described [41]. Isolated total lung cells were *in vitro* re-challenged with αPD-1 (10 μg/ml) or IgG2a (10 μg/ml) or a combination of both for 24 h at 37°C, 5%CO_2_.

### Hematoxylin and eosin (H&E) staining of tumor bearing murine lungs

Tumor bearing lungs were removed and fixed in 10% formalin (diluted in PBS) followed by dehydration and then embedded in paraffin. 5μm lung sections cut from the paraffin block were stained with hematoxylin and eosin in order to detect the tumor infiltrated areas.

### May-Gründwald-Giemsa staining

Cytospins of total cells of the bronchoalveolar lavage fluid were stained with the May-Grünwald-Giemsa solution (Carl Roth, Karlsruhe, Germany) according to the manufacturer’s protocol.

### Flow cytometric analysis of murine cells

Total single lung cell suspension was prepared as previously described [41]. Total lung cells were incubated with the respective mix of surface antibodies diluted in PBS and incubated for 30 min at 4°C in the dark. For intracellular staining lung cells were fixed and permeabilized using the Fixation/Permeabilization Concentrate/Diluent in accordance to the manufacturer’s protocol (eBioScience, San Diego, CA, USA). For cytokine staining total lung cells were cultured for 24 h in RPMI-1640 medium (Gibco) supplemented with 10% FCS, 1% L-Glut, 1% Pen/Strep, in the presence of 10 μg/ml αCD3 and 1 μg/ml αCD28 at 37°C, 5%CO_2_. Lung cells were stimulated for 4 h with ionomycin (1 μM, Sigma-Aldrich), PMA (50 ng/ml, Sigma-Aldrich) and Golgi inhibitor monensin (2 μM, eBioScience). Antibodies for intracellular staining were diluted in Permeabilization Buffer (eBioScience) and incubated for 30 min, at 4°C in the dark. Flow cytometric analysis was performed with a FACS Calibur and a Canto II (BD BioScience) at the Molecular Pneumology Department. Data were examined by Cell Quest 4.02 (BD BioScience), FACS Diva (BD BioScience) and Flow-Jo 10.2 (FlowJo, LLC, Oregon, USA). Following antibodies were used for flow cytometric analysis: αCD3 (1/200, FITC, 17A2, BD BioScience), αCD4 (1/200, FITC, H129.19, BD BioScience), αCD4 (1/200, Alexa Fluor 647, RM4-5, BD BioScience), αCD8 (1/400, APC, 53-6.7, BD BioScience), αCD8 (1/200, PerCp, 53.6-7), αCD11β (1/200, APC, M1/70, BD BioScience), αCD25 (1/100, PerCpCy5.5, PC61, BD BioScience), αPD-1 (1/200, APC, J43, BD BioScience), αPD-L1 (1/200, PE, MIH5, BD BioScience), αF4/80 (1/200, PerCp, BM8, BD BioScience), αTNFR1 (1/100, PE, HM104, ThermoFisher), αTNF R Type II (1/100, PE, TR75-89, BioLegend), αTbet (1/20, PE, O4-46, BD BioScience), αFoxp3 (1/20, APC, 3G3, Miltenyi Biotec), αTNFα (1/200, APC, Mab11, BD BioScience), αEomes (1/150, e-Fluor780, eBioScience).

### Apoptosis assay

10^5^ cells were harvested and stained with Annexin V FITC (BD, BioScience) and Propidium iodide (BD, BioScience) dissolved in 1× Annexin V Binding Buffer (BD, BioScience). After 15 min incubation in the dark at room temperature (RT), apoptosis assay was analyzed by using FACS Calibur and FACS CantoII (BD BioScience, Franklin, Lakes, USA). Data sets were analyzed by Flow-JoV10 (FlowJo, LLC, Oregon, USA).

### Generation of bone marrow-derived macrophages (BMDM) and M1 differentiation assay

Femur and tibia were dissected and cleaned from muscles without injuring the bone. Bones were sterilized in 70% ethanol. Bones were opened at their poles and bone marrow cells were flushed out with RPMI-1640 (Gibco) using a 20 Gauge needle. Cells were centrifuged at 300 g, 10 min at 4°C and resuspended in 5 ml ammonium-chloride-potassium (ACK) lysis buffer in order to lyse erythrocytes. Cells were centrifuged at 1500 rpm, 5 min and 4°C and washed with RPMI-1640 (Gibco) supplemented with 10% FCS. After another centrifugation step (1500 rpm, 5 min, 4°C) cells were seeded in BMG-medium RPMI 1640 supplemented with 10% FCS, 1% Pen/Strep, 1% L-Glut, 1% NEA-Mix, 1% Sodium pyruvate, 5% Panexin, 2% β-Mercaptoethanol and 0.05% recombinant M-CSF (BioLegend, Eching, Germany) and plated on 6 cm petri dishes. On day 4 and 8 medium was changed to remove floating cells. For macrophage differentiation assay, samples were detached on day 11 and stimulated with Lipopolysaccharide (LPS, 0.1 μg/ml, Sigma-Aldrich) and IFNg (0.01 μg/ml, BioLegend) for 48 h at 37°C, 5% CO_2_ and then harvested for qPCR analysis.

### Isolation of murine CD8^+^ and CD11b^+^ cells from naïve or tumor bearing lungs

Single cell suspension from tumor bearing or naïve murine lungs were prepared as previously described in [41]. CD8^+^ cells were isolated from tumor bearing or naïve lungs by magnetic cell separation using CD8a (Ly-2) MircoBeads, mouse kit (Miltenyi Biotec) in accordance to the manufacturer’s instructions. CD11b^+^ cells were isolated from lungs from tumor bearing mice using the CD11b MicroBeads, human and mouse kit (Miltenyi Biotec) according to the manufacturer’s instructions. Isolated cells were cultured in RMPI-1640 supplemented with 10% FCS, 1% L-Glut and 1% Pen/Strep in the presence of αCD3 (10 μg/ml, BD BioScience) and αCD28 (1 μg/ml, BioLegend). After 24 h incubation at 37°C, 5% CO_2_ supernatants were collected and used for Cell Viability Assay.

### Statistical analysis

Statistics were performed with Graph-Pad Prism7. The unpaired *t*-test was used for parametric data containing no more than two groups. Linear regression analysis was used for correlation analysis. Data are presented as mean ± SEM and significance levels indicated as follows: ^*^p ≤ 0.05, ^**^p ≤ 0.01, ^***^p ≤ 0.001.

## SUPPLEMENTARY MATERIALS FIGURES


