# Supplementary material for: STAT1 deficiency supports PD-1/PD-L1 signaling resulting in dysfunctional TNFα mediated immune responses in a model of NSCLC
Source: Oncotarget. 2018 Dec 14;9(98):37157–72. doi: 10.18632/oncotarget.26441 (PMC6324686; doi:10.18632/oncotarget.26441)
Supplement: Supplementary file 1 [file oncotarget-09-37157-s001.pdf]

STAT1 deficiency supports PD-1/PD-L1 signaling resulting in dysfunctional TNFα mediated immune responses in a model of NSCLC

SUPPLEMENTARY MATERIALS

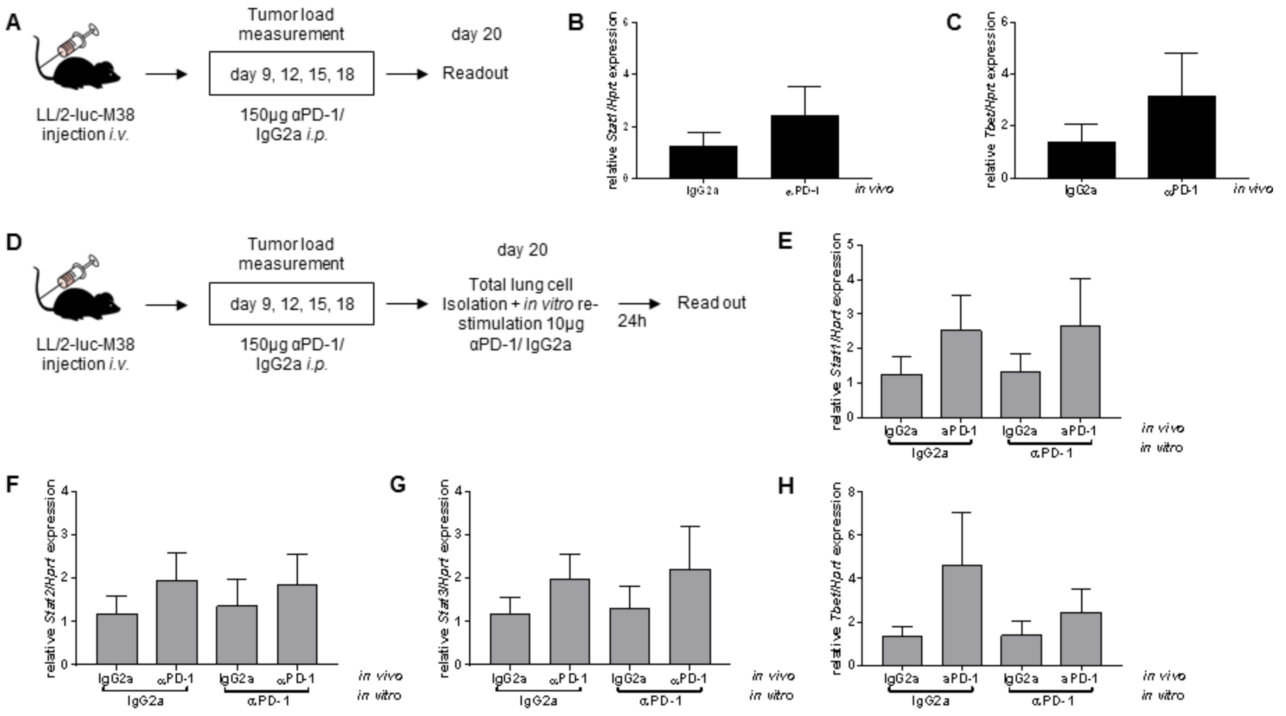

Supplementary Figure 1: αPD-1 -antibody therapy resulted in increased Stat1 mRNA expression in a murine model of lung adenocarcinoma. (**A**) Experimental design. Mice were injected i.v. with 1×10^6 LL/2-luc-M38 cells. Starting at day 9 post injection, every three days mice were treated i.p. with 150 µg αPD-1 -antibody or related isotype control IgG2a. Lung tumor development was detected via bioluminescence. (**B**) Blocking of PD-1 *in vivo* resulted in an tendencial upregulation of Stat1 mRNA expression total lung cells derived from tumor bearing mice compared to the related IgG2a isotype control (nIgG2a = 4; nαPD-1 = 4). (**C**) *In vivo* blockade of PD-1 is associated with increasing Tbet mRNA expression level in total cells isolated from tumor bearing mice compared to tumor bearing mice treated with the related isotype control (nIgG2a = 4; nαPD-1 = 4). (**D**) Experimental design of *in vitro* re-challenge. At day 20 post injection, total lung cells were *in vitro* re-challenged with αPD-1, related isotype control IgG2a, a combination of both antibodies or left untreated (unstim). (**E**) After *in vitro* re-challenge with αPD-1 or isotype control IgG2a for 24 h, Stat1 mRNA expression was tendencially upregulated in total lung cells isolated from tumor bearing mice in case of αPD-1 blockade *in vivo* (nIgG2a = 4; nαPD-1 = 4). (**F**) *In vitro* re-challenge with αPD-1 antibody or IgG2a showed slightly increased Stat2 mRNA expression in total lung cells (nIgG2a = 4; nαPD-1 = 4). (**G**) After *in vitro* re-challenge with αPD-1 antibody or IgG2a, Stat3 mRNA expression of total lung cells isolated from tumor bearing mice with *in vivo* PD-1 blockade revealed a tendencial increase (nIgG2a = 4; nαPD-1 = 4). (**H**) *In vitro* re-challenge with αPD-1 -antibody or IgG2a did not affect the upregulation of Tbet mRNA expression during *in vivo* blockade with αPD-1 antibody in total lung cells derived from tumor bearing mice (nIgG2a = 4; nαPD-1 = 4). Data are presented significantly as mean values ± SEM; no significances with unpaired *t*-test.

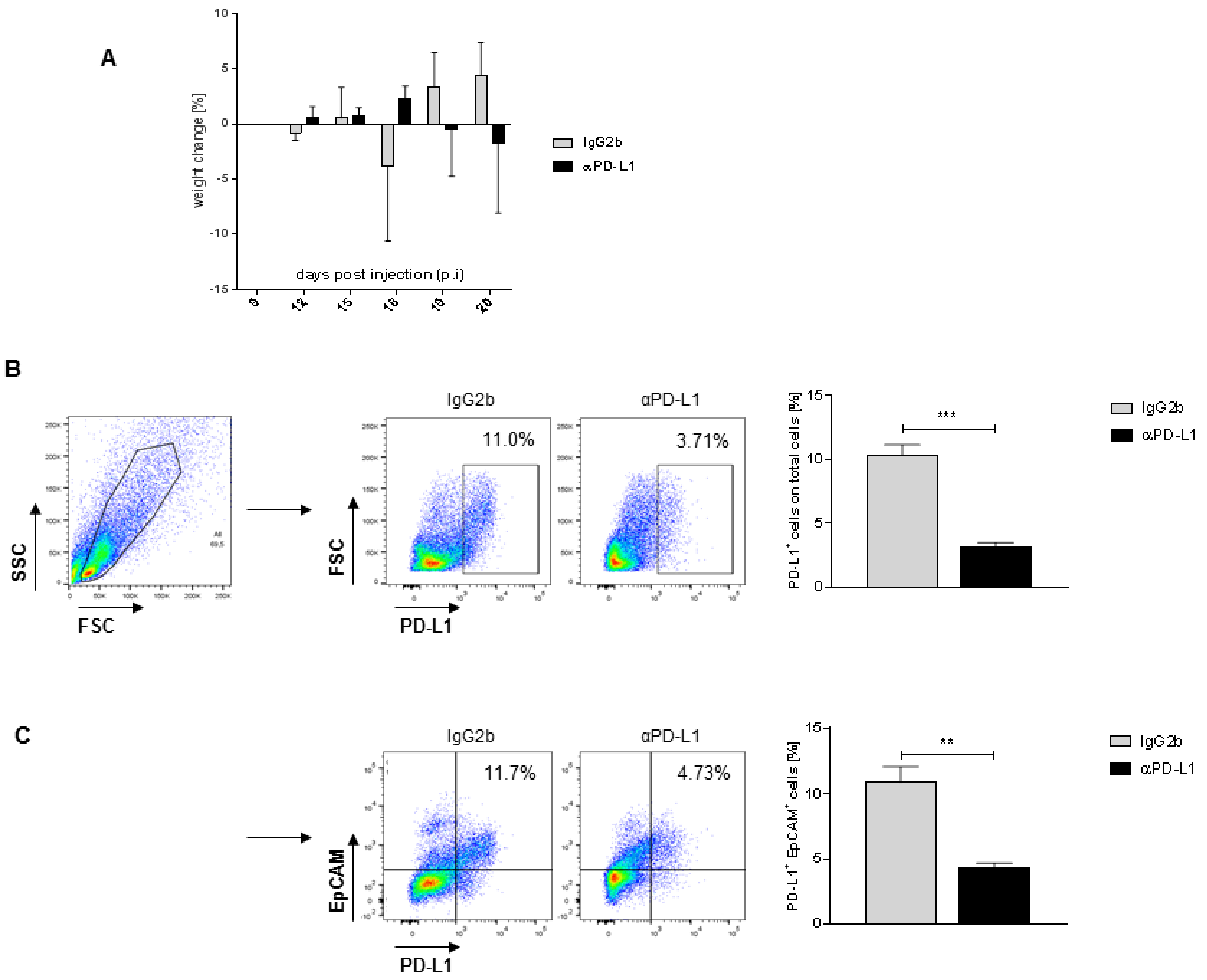

Supplementary Figure 2: Immuno-checkpoint blockade of PD-L1 antibody in a murine model of lung adenocarcinoma. (**A**) αPD-L1 -antibody treatment significantly inhibited the expression of PD-L1 on total lung cells derived from tumor bearing mice compared to mice treated with the related isotype control (nIgG2b = 3; nαPD-L1 = 4). (**B**) Significant downregulation of PD-L1+ EpCAM+ cells in tumor bearing lungs from mice treated with αPD-L1 compared to mice treated with isotype control (nIgG2b = 3; nαPD-L1 = 4). (**C**) Tumor bearing mice treated with αPD-L1 -antibody did not show a significant weight change compared to tumor bearing mice treated with the related isotype control IgG2b (nIgG2b = 3; nαPD-L1 = 4). Data are presented as mean values ± SEM; unpaired *t*-test $p < 0.05^{*}$, $p < 0.01^{**}$, $p < 0.001^{***}$.

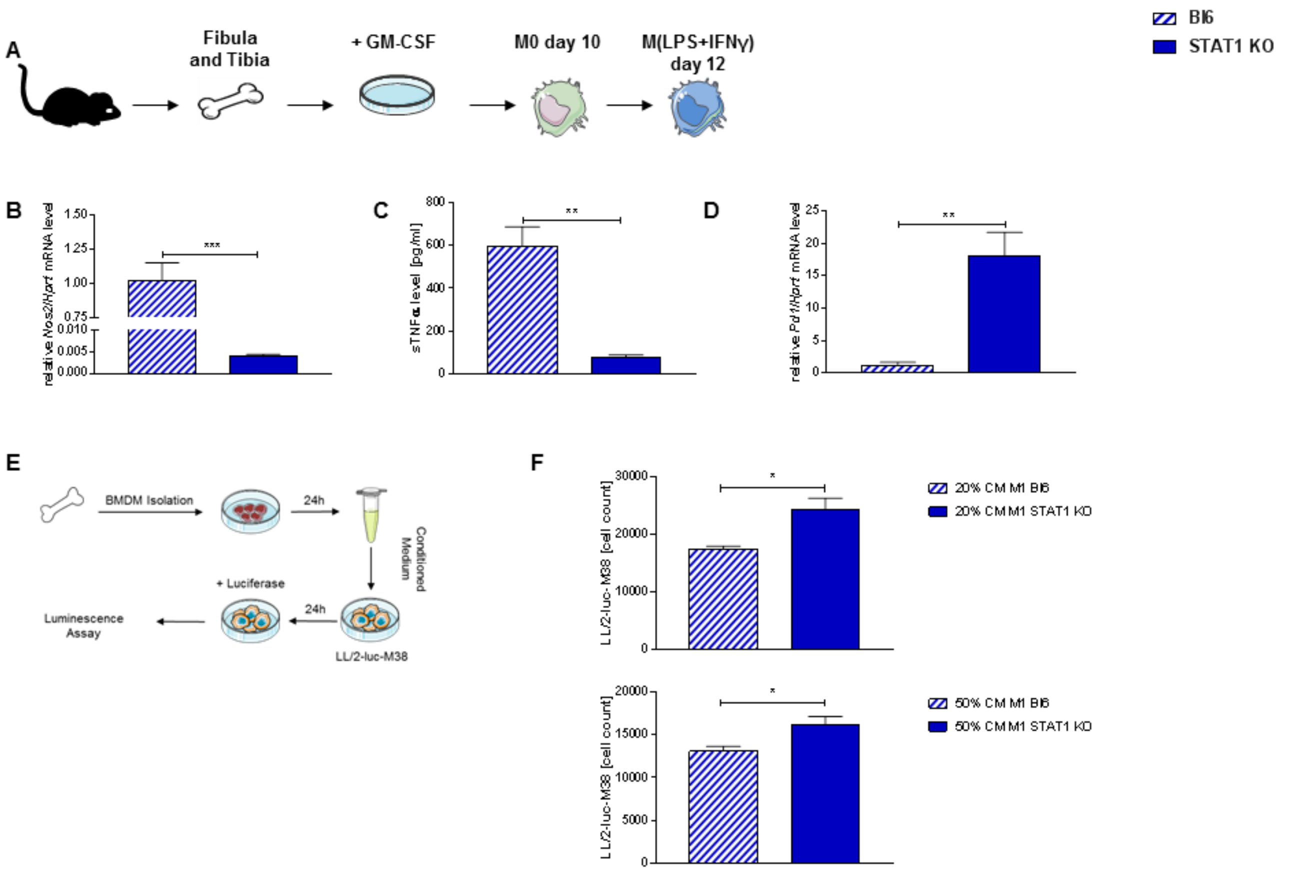

Supplementary Figure 3: STAT1 is necessary for the ability of M1-like macrophages to reduce cell viability of LL/2-luc-M38 lung adenocarcinoma cells. (**A**) Experimental design for the differentiation assay of M1 (LPS+IFNγ) macrophages derived from the bone marrow. Bone marrow derived macrophages (BMDM) were isolated from fibula and tibia and cultured with GM-CSF for ten days. M1 differentiation was induced via stimulation with exogenous LPS and IFNγ for 48 h. (**B**) Strong reduction of Nos2 mRNA expression in naïve STAT1 KO mice compared to naïve Bl6 mice (nBl6 = 8; nSTAT1 KO = 8). (**C**) Reduced secretion of soluble TNFα from Stat1 deficient M1-like macrophages compared to naïve Bl6 M1 macrophages (nBl6 = 8; nSTAT1 KO = 8). (**D**) Dramatic increase of Pd1 mRNA on Stat1 deficient M1 macrophages compared to M1 macrophages derived from Bl6 mice (nBl6 = 8; nSTAT1 KO = 8). (**E**) Experimental design of luminescence cytotoxic assay. LL/2-luc-M38 cells were incubated with 20% or 50% of supernatants derived from M1 macrophages diluted in DMEM plus supplements (conditioned medium; CM) for 24 h. Living LL/2-luc-M38 cell count was determined by luminescence analysis. (**F**) Increased cell viability of LL/2-luc-M38 cells was detected when incubated with M1-like macrophages derived from the bone marrow of STAT1 KO mice compared to M1 macrophages derived from Bl6 mice (nBl6 = 8; nSTAT1 KO = 8). Data are presented as mean values ± SEM; unpaired *t*-test $p < 0.05^{*}$, $p < 0.01^{**}$, $p < 0.001^{***}$.

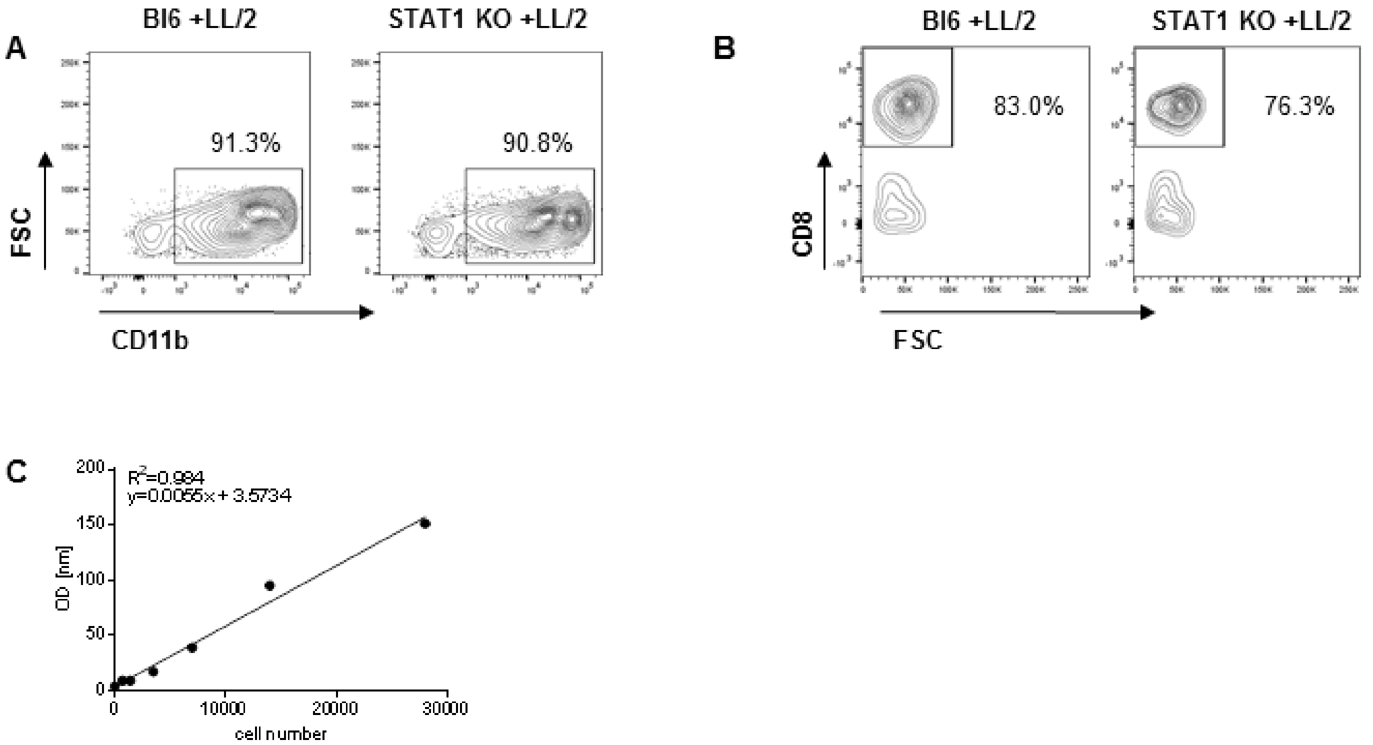

Supplementary Figure 4: Additional information on the cytotoxicity assay with conditioned medium derived from isolated CD11b or CD8 cells isolated from tumor bearing mice. (**A**) CD11b+ cells were isolated via MACS beads. Purity was verify by flow cytometric analysis. Purity of CD11b+ cells from tumor bearing Bl6 and STAT1 KO mice was about 90%. (**B**) CD8+ T cells were isolated via MACS beads. Purity was determined by flow cytometric analysis. CD8 purity from tumor bearing Bl6 or STAT1 KO mice was about 80%. (**C**) 0, 700, 1400, 3500, 7000, 14000 and 28000 LL/2-luc-M38 cells were seeded as a standard curve in order to determine the cell growth and viability.

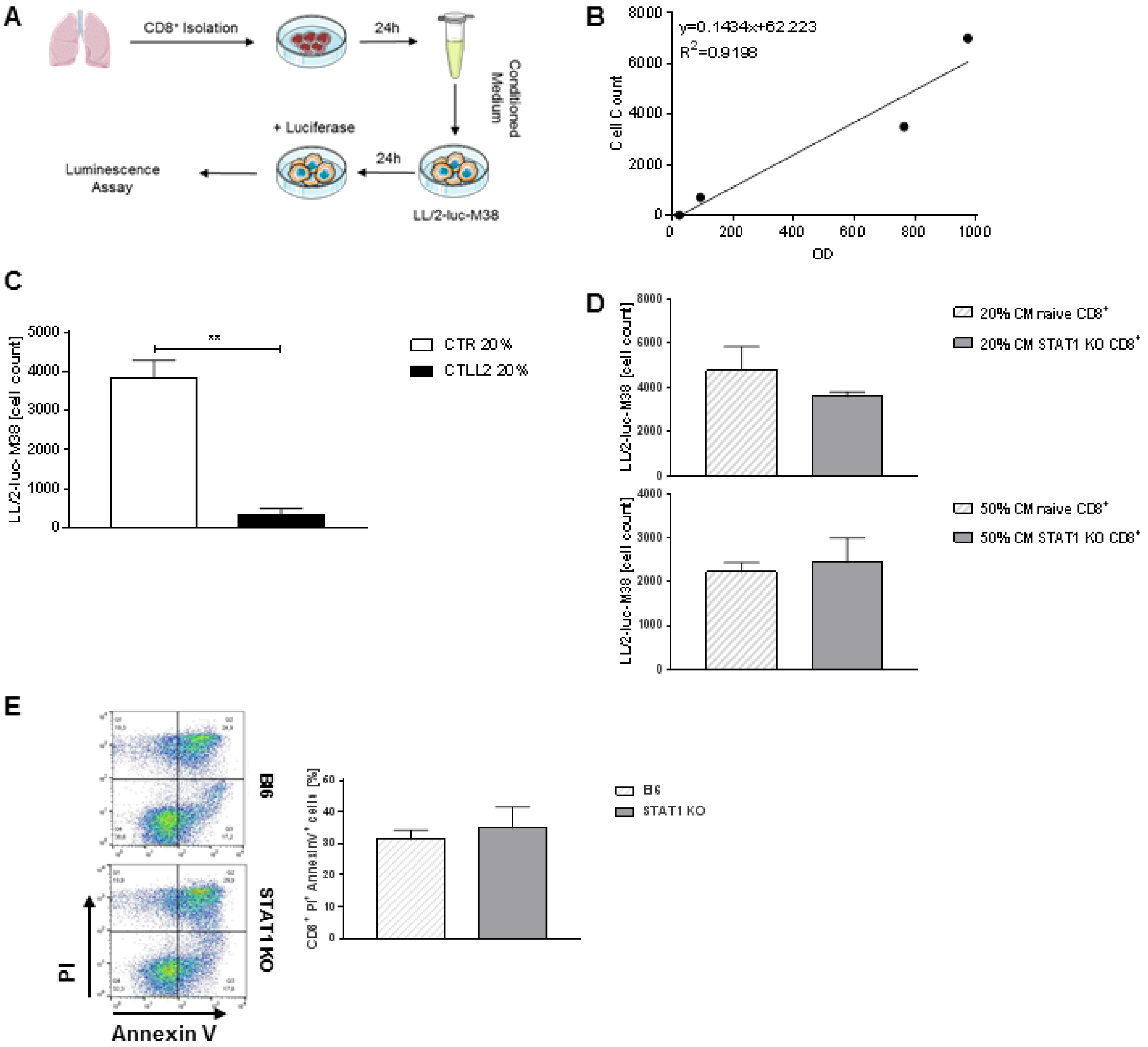

Supplementary Figure 5: Stat1 deficiency did not impair cytotoxic capacity and viability of CD8+ T cells in naïve mice. (**A**) Experimental design. CD8+ cells were isolated via MACS beads from total lung cells. Supernatant was diluted with RPMI with supplements (conditioned medium = CM). LL/2-luc-M38 lung adenocarcinoma cells were co-cultured for 24 h with CM from Bl6 or STAT1 KO mice. LL/2-luc-M38 cell viability was determined via bioluminescence analysis. (**B**) 0, 700, 1400, 3500, 7000, 14000 and 28000 LL/2-luc-M38 cells were seeded to determine a standard curve. (**C**) CM derived from CTLL2 cells were used as a positive control. (**D**) Stat1 deficiency did not impact the cytotoxic activity from isolated lung CD8+ T cells compared to cytotoxic activity from CD8+ lung T cells derived from naïve Bl6 mice (nBl6 CD8+ T cells = 5; nSTAT1 KO CD8+ T cells = 5). (**E**) Stat1 deficiency had no impact on the apoptotic behavior of CD8+ T cells isolated from total lung cells compared to CD8+ lung T cells derived from Bl6 mice (nBl6 CD8+ T cells = 5; nSTAT1 KO CD8+ T cells = 5). Data are presented as mean values ± SEM; unpaired *t*-test $p < 0.05^{*}$, $p < 0.01^{**}$, $p < 0.001^{***}$.

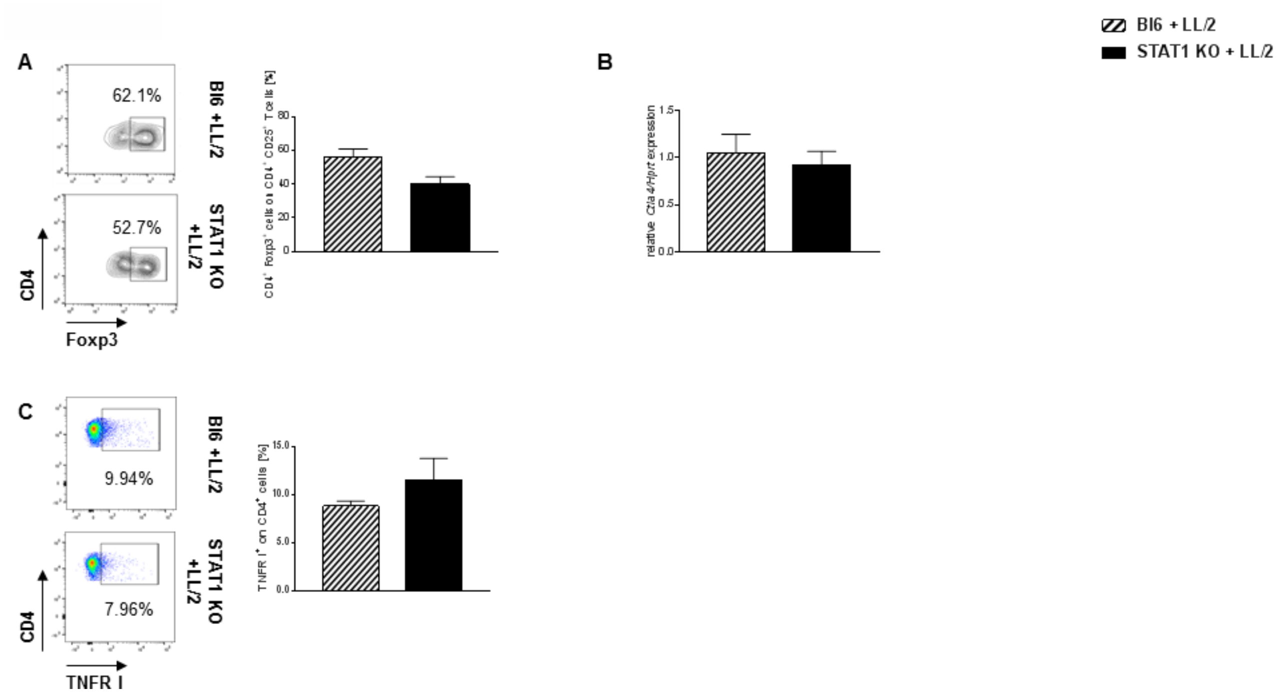

Supplementary Figure 6: Stat1 deficiency did not affect Foxp3+ Tregs or Ctla4 mRNA expression. (**A**) Stat1 deficiency did not affect the accumulation of Tregs, characterized as Foxp3+ previously gated on CD4+ CD25+ T cells compared to tumor bearing wild-type mice in a murine model of lung adenocarcinoma (nBl6 + LL/2 = 5; nSTAT1 KO + LL/2 = 4). (**B**) No difference of Ctla4 mRNA expression was detected in total cells isolated from tumor STAT1 KO mice or Bl6 mice (nBl6 + LL/2 = 5; nSTAT1 KO + LL/2 = 4). (**C**) Stat1 deficiency had no impact on the expression of TNFR I on lung tumor infiltrating CD4+ T cells compared to the expression of TNFR I on CD4+ T cells derived from Bl6 mice (nBl6 + LL/2 = 7; nSTAT1 KO + LL/2 = 7). Data are presented as mean values ± SEM; unpaired *t*-test $p < 0.05^{*}$, $p < 0.01^{**}$, $p < 0.001^{***}$.
